# MatplotAlt: A Python Library for Adding Alt Text to Matplotlib Figures in Computational Notebooks

**DOI:** 10.1111/cgf.70119

**Published:** 2025

**Authors:** Kai Nylund, Jennifer Mankoff, Venkatesh Potluri

**Affiliations:** 1 Paul G. Allen School of Computer Science & Engineering, University of Washington; 2 University of Michigan School of Information

## Abstract

We present MatplotAlt, an open-source Python package for easily adding alternative text to Matplotlib figures. MatplotAlt equips Jupyter notebook authors to automatically generate and surface chart descriptions with a single line of code or command, and supports a range of options that allow users to customize the generation and display of captions based on their preferences and accessibility needs. Our evaluation indicates that MatplotAlt’s heuristic and LLM-based methods to generate alt text can create accurate long-form descriptions of both simple univariate and complex Matplotlib figures. We find that state-of-the-art LLMs still struggle with factual errors when describing charts, and improve the accuracy of our descriptions by prompting GPT4-turbo with heuristic-based alt text or data tables parsed from the Matplotlib figure.

## 1. Introduction

Computational notebooks centralize code, natural language, and visualizations in a single interactive medium. Because of their versatility and ease of use, Jupyter notebooks have become popular for teaching, communicating results and conducting analysis. Despite their ubiquity, notebooks remain inaccessible to blind and visually impaired (BVI) users due to the authoring practices, tools, and infrastructures used in their creation and sharing [PSTM23]. Notably, in their analysis of 100,000 notebooks, Potluri and Singanamalla *et al.* found that 99.81% of programmatically generated images did not have associated alternative text [PSTM23], which is a critical accessibility barrier [EBM22, C*08].

From a sample of 10 million notebooks, the JetBrains Datalore team found that about 42% had programatically generated images [Guz]. The vast majority of these were found to be data visualizations created with the Matplotlib or Seaborn libraries, neither of which contain methods to easily embed image descriptions or alternative text [PSTM23, Hc19]. Other visualization tools like Altair [VGH*18] are beginning to include options to create accessible text structures for screen reader users, but these updates are not compatible with most inaccessible notebooks on the web that do not use these libraries [Guz]. Additionally, no popular computational notebook software currently supports the addition of descriptive text to displayed images.

To help notebook users create and consume image descriptions for the majority of notebooks, we present *MatplotAlt*, a Python package to add alt text to Matplotlib figures. MatplotAlt provides functions to automatically generate descriptions from a Matplotlib figure object, and several methods to embed and export its alt text. These automatically generated captions are informed by Lundgard and Satyanarayan’s four-level model of semantic content [LS21], a set of guidelines to effectively describe data visualizations. To create both accurate and fluent chart descriptions, MatplotAlt provides two options for generating alt text: a heuristic based approach which directly uses figure attributes and data, and a vision-language model (VLM) based method which takes the full image as input. While state-of-the-art VLMs can generate relevant alt text [CLD*23,ZW24, SWB24], we confirm that they are still prone to factual errors on both simple univariate and complex Matplotlib figures. We show that prompting VLMs with template-based alt text is a simple way to increase their accuracy and similarity to human-written descriptions. In this work, we contribute:

- MatplotAlt, the first Python library to support programmatic generation, inclusion, and dissemination of customizable alternative texts for Matplotlib images in computational notebooks.
- Quantitative and qualitative evaluations of MatplotAlt’s methods for generating alt text, including a detailed analysis of errors in VLM descriptions.
- Strategies to improve the quality of VLM descriptions for Matplotlib figures.

MatplotAlt’s integration into the notebook pipeline, ease of use, and readiness for VLM-generated descriptions have the potential to make notebooks accessible to BVI users at scale and serve as a blueprint for plotting libraries to build in native support for alt text. The scenarios below show how MatplotAlt can help users perceive Matplotlib images and work with notebooks. We make Matplotalt and our evaluation datasets publicly available at https://github.com/make4all/matplotalt.

### 1.1. Example Scenarios

Aida is a BVI developer in a data analysis team comprised of BVI and sighted scientists. Aida wants to visualize data from their research project using Matplotlib in a Jupyter notebook, then add alt text to their figures for future screen reader users and their own reference. To include automatically generated alternative text in the HTML output of the notebook, they replace calls to matplotlib.pyplot.show with MatplotAlt’s show_with_alt function. Rory, Aida’s sighted colleague adds additional analysis that results in complex figures. Rory uses generate_alt_text, which returns generated alt text for the last figure as a string. They check for factual correctness and manually add their own context and insights to the returned string. Finally, Rory displays their customized alt text with add_alt_text for Aida to review.

Dez is a BVI student trying to learn a new Python package by reading its documentation page. Using a screen reader, they notice most of the examples are in a computational notebook with several Matplotlib figures. Unfortunately, the only text read when they select one of the images is “*No description has been provided for this image*”, which is the default in Jupyter. Dez downloads the notebook and runs MatplotAlt’s alttextify command from the terminal to automatically create and embed descriptions for each figure. After reading the generated alt text, Dez feels several figures are still lacking context, so they rerun alttextify with a VLM by passing an API key. Once Dez has read both versions of figure alt text they have a clearer idea of how to use the library in their coursework. Satisfied with this output, Dez reruns alttextify with the -s new_cell argument to store generated alt text as a code comment. Dez converts this updated notebook into a python file using standard nbconvert tools, then continues to work on the assignments with the alt-text and starter code giving them the necessary information to use the library effectively.

These scenarios describe how the various options provided by MatplotAlt equip a BVI expert to exercise agency by generating customized alternative texts, and overcome accessibility barriers previously posed by poor infrastructure and authoring practices.

## 2. Related Work

MatplotAlt is motivated by a growing set of literature on generating descriptive alt text and the (in)accessibility of visualization systems. We provide a brief summary about accessible visualizations, motivate the need to make them accessible in the context of computational notebooks, and provide relevant background on emerging automatic description techniques.

### 2.1. Accessibility of Web Visualizations

Despite improvements in systems to generate and publish alt text, most figures on the web remain inaccessible to screen reader users. Extending the Web Content Accessibility Guidelines (WCAG), Elavsky *et al.* developed Chartability, a set of heuristics to evaluate the accessibility of data visualizations, including for keyboard navigation, screen reader inspection, and cognitive barriers [EBM22]. Their work highlights the changing nature of accessibility standards, and the need for context-specific guidelines in visualization.

Visualizations on the web rarely adhere to these guidelines or contain even basic accessibility support necessary for BVI users to gain insights from data. BVI users report several common pain points, including invisible or incomprehensible figures, a lack of description of trends and axes, and the inability to expose data in tables and arrays [SCWR21]. Visualization dashboards, which are often presented as web interfaces, also frequently contain barriers like inconsistent semantic structure and unsurfaced changes [SHHM23].

These barriers are compounded by a lack of practical methods for creating accessible outputs. Joyner *et al.* survey visualization practitioners and identify complex / interactive figures and lack of accessibility support in visualization tools as obstacles preventing the creation of accessible figures [JRG*22]. Several systems aim to make creating alt text easier [MCLM21, SWB24, MFM*24]. Mack *et al.* [MCLM21] explore using a template interface for authoring figure descriptions in PowerPoint with separate boxes to describe subjects, interactions, and other features. Singh *et al.* support authoring alt text for scientific publications with an interface that extracts figures, captions, and data from uploaded PDFs, then provides appropriate guidelines and suggestions generated by a large language model (LLM) [SWB24]. They find that most users prefer the extra assistance provided by templates, and that they help users know what to include in alt text [MCLM21]. Singh *et al.* also find that LLM-generated alt texts were a useful starting point for most users [SWB24]. These results inform our decision to support automatically generating starter alt text using either a template (with no user input) or VLM. MatplotAlt extends the capabilities of this growing body of work to Python figures in notebooks, allowing us to more easily extract data and features from programmatically generated charts, and address the specific accessibility barriers of this environment.

Guidelines for describing figures have also improved in the last three years. Lundgard and Satyanarayan define a theory-grounded four-level model of semantic content for describing data visualizations [LS21]. Different levels include information about encodings and axes (level 1), relevant statistics (level 2), trends (level 3), and broader context about the chart (level 4). MatplotAlt uses these guidelines to generate descriptions depending on users’ desired level of detail.

### 2.2. Automatically Generating Alt Text

Several systems focus on automatically generating accessible figures from data using heuristics and templates. Mirri *et al.* explore using CSV content to create screen reader compatible XML graphics including basic alt information [MPS* 17], and Sharif *et al.* develop a jQuery plugin which summarizes HTML/Json figure types, values, and basic statistics [SF18]. One limitation of alt text created from static templates alone is their lack of interactivity [SCWR21]. Allowing a range of exploration methods, BrailleR [GWS*24] supports both creating templated textual descriptions of graphs in R and exploring different chart features with a screen reader in an interactive SVG. MatplotAlt aims to similarly support exploring charts in Python notebooks with both templated and VLM descriptions.

Recently, there has been growing interest in generating captions and interactive descriptions for data visualizations using vision transformers. MatCha [LPK*22] and UniChart [MKD*23], for instance, are both pretrained image-to-text Transformer [VSP* 17] models to automatically summarize and respond to user questions about figures. ChartVLM [XZY*24] improve on chart QA tasks by scaling up their model and using a classifer to transform natural language instructions into a discrete set of tasks.

Though these efforts demonstrate the viability of vision transformer models to generate chart captions and support conversational data analysis, they do not account for the nuanced information needs of BVI experts. To address this gap, Tang *et al.* curate Vis-Tex [TBS23], a dataset of image / caption pairs adhering to the four-level semantic model, which is informed by preferences and needs of BVI users. VLMs are also prone to errors that can mislead users relying on these descriptions [ZW24, KLL*22]. Tang *et al.* qualitatively analyze errors produced by their models and find that approximately half of generated statements were factually incorrect [TBS23]. Using LLMs to generate alt text can also create new barriers like API costs, large downloads, and knowledge of natural language processing libraries.

At the same time, heuristic-based alt text is prone to repetition over multiple charts and may be unable to capture context that is essential to interpreting figures. These limitations motivate our decision to provide options to generate both heuristic and VLM-based alt text. For high-stakes analysis in the medical domain, for example, MatplotAlt’s dry but interpretable templated descriptions are likely more appropriate [Rud19] compared to those generated by a VLM that potentially contain errors and hallucinations. Descriptions for personal learning on well-documented problems, however, may benefit from the adaptability and extra context provided by VLMs [RNK*23].

### 2.3. Accessibility of Computational Notebooks

Despite their ubiquity, computational notebooks remain inaccessible to BVI users. Computational notebook usage is growing, with the number of .ipynb files on Github increasing from 200,000 in 2015 to 2.5 million in 2018 [Per18], and a dataset of 10 million Jupyter notebooks released in 2020 [Guz]. Several authors have even suggested making notebooks the primary artifact of scientific publications [Som18, RMABR20, CSR*23]; but fundamental accessibility barriers remain unaddressed. Building on prior work documenting pain points in computational notebooks [CPH*20], Potluri *et al.* analyze the inaccessibility of Jupyter for BVI developers and users [PSTM23]. In their evaluation of 100,000 notebooks, they found that 99.81% of programmatically generated images, most of which were created with Matplotlib or seaborn, lacked associated alt text. They also identify a range of common WCAG failures in notebooks including low color contrast, incorrectly nested headers, and a lack of correctly formatted data tables.

Based on these errors, the authors provide a set of guidelines for improving the accessibility of Jupyter notebooks. These include using the four-level semantic model to generate and include alt text for plotting libraries like Matplotlib using the figure object, and automatically generating markdown tables for applicable visualizations. MatplotAlt is directly inspired by these recommendations, with the goal of providing an easy interface to include alt text and data tables for figures in Jupyter notebooks.

## 3. The MatplotAlt System

**Figure 2:**
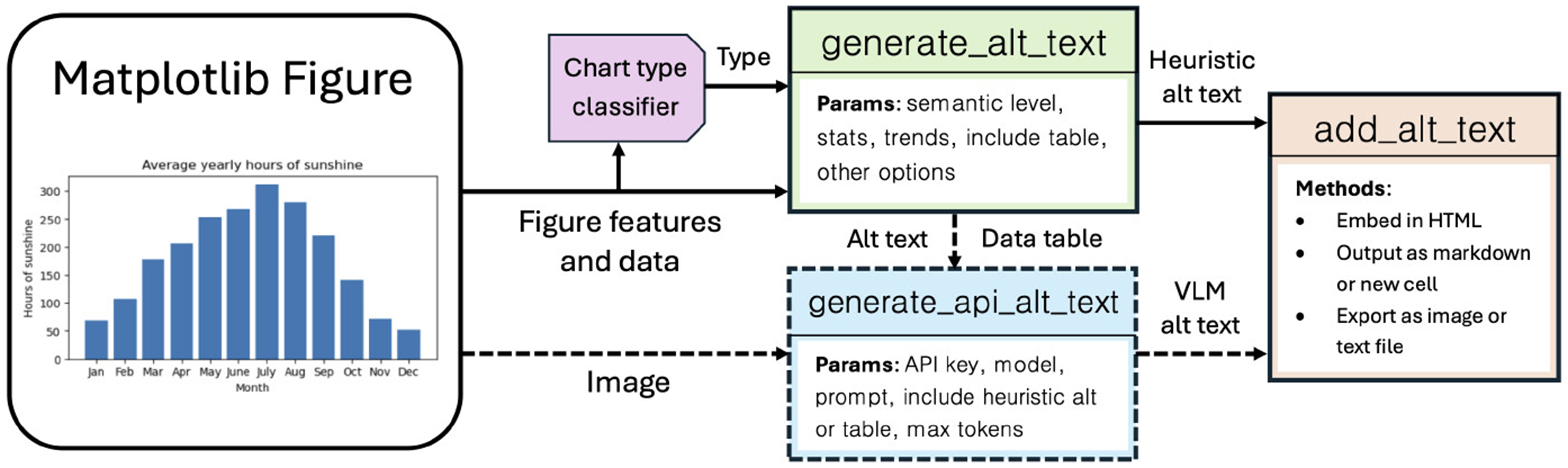
The MatplotAlt system. Heuristic-based alt text of a given semantic level is generated from Matplotlib figure attributes using the generate_alt_text function. Alternatively, VLM-based descriptions can be generated with the figure as input using generate_api_alt_text. Descriptions are then embedded in the notebook or exported using add_alt_text.

We describe the algorithms, templates, and libraries we use to automatically generate and surface alt text for Matplotlib figures. Figure 2 depicts a simplifed view of the MatplotAlt system highlighting components responsible for parsing chart features (§3.1), generating heuristic (§3.2) and VLM-based (§3.3) alt text, and surfacing descriptions for screen readers (§3.4). To ensure that accessibility barriers posed by notebook software do not hinder the utility of MatplotAlt, we also provide options to use these tools outside the notebook environment (§3.5).

### 3.1. Inferring Chart Data and Features

Chart descriptions should contain information about the chart type, and other visual encodings to adhere to the four-level model of semantic content [LS21]. To generate alt text that includes these details, we parse most features and data directly from Matplotlib figures. To get a list of the tick labels on the x-axis, for example, we call Matplotlib’s get_xticklabels() method on the most recently plotted figure. Chart type is one notable exception, as Matplotlib only stores abstract elements like rectangles, lines, and points. MatplotAlt infers type based on these components. If a plot has multiple point objects without any lines, for instance, we label it as a scatterplot. If the figure contains both lines and points, then we infer that it is a line plot. And if the plot contains a quadmesh, it is likely a heatmap or image. In addition to chart features, we also consider how data is internally represented when determining type. Heatmaps and images, for example, contain 2d arrays of values while line plots have an iterable of line objects and values.

One downside of this approach is that it assumes all charts have a single type. For example, our current system cannot correctly classify overlaid bar and line plots in the same figure. Our system also currently fails on interactive and dynamically updated charts. Unlike more complex classification models, however, MatplotAlt can be easily extended to new chart types by adding checks for new attributes without the need for retraining. Extending previous work [KLL*22, TBS23] that focus on only the one to three most popular chart types, MatplotAlt currently supports ten types of figures: *line, bar, scatter, radial line, pie, strip, contour, heatmap, image*, and *boxplot*.

### 3.2. Generating Heuristic-based alt text

To automatically generate alt text for Matplotlib figures using templates and heuristics, MatplotAlt provides the generate_alt_text function. Like our chart type detection, information used in descriptions is extracted directly from the most recent Matplotlib object.

Users can specify the amount of detail to include in alt text through the desc_level parameter, based on L1-L3 semantic levels [LS21]:

*L1:* Alt text includes the chart’s type, title, color encodings, annotations, and the scale and range of each axis.

*L2:* Includes L1 plus statistics for each variable. We start with the list of statistics mentioned in the four-level model of semantic content (extrema, outliers, and correlations), and expand to several other easily computed metrics like standard deviation and median. We also adjust the default supported statistics for different chart types. Alt text for scatter plots, for instance, will include minima, maxima, and a line of fit; boxplots’ will contain interquartile ranges; and contours’ will contain the center point of the min/max contour. These defaults were chosen both based on commonly performed analyses (e.g., line of fit) and feasibility (e.g., boxplots may not display individual points). Users can manually specify which stats to include through the stats parameter.

*L3:* Includes L2 plus chart trends such as increasing / decreasing patterns if applicable and the stability (e.g., “fluctuating” vs. “strictly increasing”) of each variable. MatplotAlt currently only supports trends for contiguous two-dimensional data. No L3 description is added to scatter, strip, box, and contour plots. Like, stats, users can specify included trends with the trends parameter.

Chart summaries provide an overall picture of Matplotlib figures, but consumers of these artifacts may also want to drill down to the underlying data [SCWR21, SZRW23]. Elavsky *et al.* suggest the inclusion of tables with figures representing data, an accompaniment overwhelmingly lacking in notebooks [EBM22, PSTM23]. To support this interactivity, MatplotAlt provides the option to include the underlying chart data as a markdown table in generated alt text. To avoid overly large tables that are hard to navigate with screen readers [WWJK22], we add an adjustable cap on the max number of table rows and columns. If there are multiple subplots in a figure, heuristic alt text and tables will be generated for each separately. Supplemental material §2 includes more detail about templates used in heuristic-based descriptions. To generate L4 descriptions (including context, explanations, and insights), we explore using a VLM in the next section.

### 3.3. VLM-based alt text

MatplotAlt implements the generate_api_alt_text function to generate figure descriptions with models hosted on OpenAI, Azure, and Huggingface APIs. Like generate_alt_text, users can specify the semantic level of VLM alt text with the desc_level parameter. Prompts to the language model, documented in Supplemental §3, include a call to describe figure details based on the given desc_level, and two example L1-L4 figure descriptions. In addition to L1-L3 alt texts, a desc_level of 4 can be passed to generate_api_alt_text. Prompts to generate L4 captions will also include the line “If possible, briefy explain domain-specific insights, current events, and socio-political context that explain the data.” Unlike heuristic alt text, we note that there is no guarantee VLM descriptions will adhere to semantic guidelines.

To capture both perceptual phenomena (e.g. overlapping points, color patterns) in images and semantic relationships between figure elements, we add chart data to prompts by including either a markdown data table (VLM + table), the heuristic-based alt text for the chart (VLM + heuristic), or both (VLM + table + heuristic). We provide examples of alt text generated using each method in supplemental material Table 1, and evaluate each method in §5.

### 3.4. Surfacing alt text in Jupyter notebooks

To make figure descriptions visible to screen reader users in Jupyter notebooks, MatplotAlt provides the add_alt_text function. Several options are supported through the methods parameter:

- “html”: displays the last figure in html with an alt property containing the given text. This is the default option in MatplotAlt, allowing authors and readers to embed and consume image descriptions without changing the layout of the notebook.
- “markdown”: adds text in markdown to the current cell output. This method visually displays alt text alongside the figure, allowing non-screenreader users to view descriptions. Markdown data tables are surfaced only in cell or saved output.
- “new_cell”: creates a new (code) cell after this one containing the given text. This method is more disruptive to the notebook structure, but allows users to easily include the description in their code, e.g. as a string variable for further processing.
- “img_file”: saves the last Matplotlib figure as a png with the given text in its alt property. This allow users to consume only the image and description without having to navigate the inaccessible notebook environment, and enables users to create accessible visualizations for use in other projects.

For ease of use, we combine generate_alt_text and add_alt_text into a single function, show_with_alt, to both generate and surface alt text in a single line of code. We do the same for VLM alt text with the show_with_api_alt command. These functions allow users to easily replace calls to matplotlib.pyplot.show with show_with_alt to generate accessible figure outputs.

### 3.5. Using MatplotAlt outside the notebook environment

Because the notebook interface itself can be a barrier to BVI users [Tea22, Tea24], MatplotAlt also provides the alttextify command to add alt text to all Matplotlib figures in a given notebook. The command, which can be run from PowerShell on Windows or the terminal on Mac, takes a notebook path, and any other arguments supported by show_with_alt to generate alt text, then embeds or exports descriptions for each Matplotlib figure in the notebook, allowing BVI users to add consumable alt text to inaccessible notebooks they encounter without ever entering the environment.

## 4. Datasets

### 4.1. VisText Captions

We quantitatively evaluate MatplotAlt’s methods for generating L3 alt text on the VisText dataset [TBS23], which consists of pairs of univariate bar, area, and line charts, and corresponding L1-L3 descriptions written by crowdworkers. VisText also contains table and scenegraph representations of charts, which we use to reverse-engineer matplotlib code for generating each figure. We then call generate_alt_text and generate_api_alt_text to create corresponding descriptions for the dev and test splits. These Matplotlib versions of VisText figures differ from the original in a few qualities like color and bar ordering that may slightly reduce our alt text’s similarity to the crowdsourced descriptions.

We use GPT4-turbo with default temperature and a max output length of 225 tokens for all L3 VLM-generated alt text. We chose this number as the smallest that did not frequently cutoff captions for the dev set, as prior work has shown that BVI users typically prefer more concise VLM-generated captions [HXP*24]. We evaluate prompting GPT4-turbo to describe charts directly (turbo) and our methods for incorperating chart data and features into prompts (turbo + table, turbo + heuristic, and turbo + table + heuristic).

To match the semantic level of human annotations in VisText, we call all generate methods with desc_level=3. Because very few of the crowdsourced captions describe chart colors or include statistics other than minima and maxima, we disable descriptions of color encodings and limit stats to the min/max in our heuristic alt text. As a comparison, we reevaluate VisText’s pretrained VL-T5 image-only, image + data table, and image + scenegraph models on the original VisText figures. Our datasets are publicly available on MatplotAlt’s code repository.

### 4.2. Matplotlib Example Gallery Captions

Although VisText provides a structured set of figures and descriptions, its limitation to univariate bar, area, and line charts makes it unrepresentative of the range of complex visualizations in notebooks on the web. As a more challenging snapshot of Matplotlib figures “in the wild”, we use notebooks from the Matplotlib gallery (https://matplotlib.org/stable/gallery). From 500 notebooks, We call alttextify <notebook path> <output> –l 3 –s html img_file to generate and export L3 alt text for 200 applicable Matplotlib figures.

Because many of these charts have data that are not easily represented in scenegraphs or tables, we generate their corresponding descriptions using only L3 heuristic, turbo, and turbo + heuristic methods. While most VisText figures display Statista data for world events (e.g., national debt, infant mortality, company sales), the gallery figures demonstrate the effect of different Matplotlib settings and are therefore tied more closely to the chart format and image itself rather than data.

To summarize, we contribute Matplotlib versions of each of the 12441 VisText figures, a small dataset of 200 figures extracted from Matplotlib Gallery notebooks, and corresponding L3 descriptions for each image using the MatplotAlt generation methods.

## 5. Evaluation

We evaluate MatplotAlt’s alt text generation methods on the VisText and Matplotlib gallery datasets. We quantitatively measure description length (§5.1), similarity to reference VisText captions (§5.2), and similarity between image and alt text embeddings (§5.3). Next, we qualitatively categorize types of errors in MatplotAlt’s descriptions (§5.4) and compare their frequency in different generation methods (§5.5).

### 5.1. Length of Generated Alt Texts

**Figure 3:**
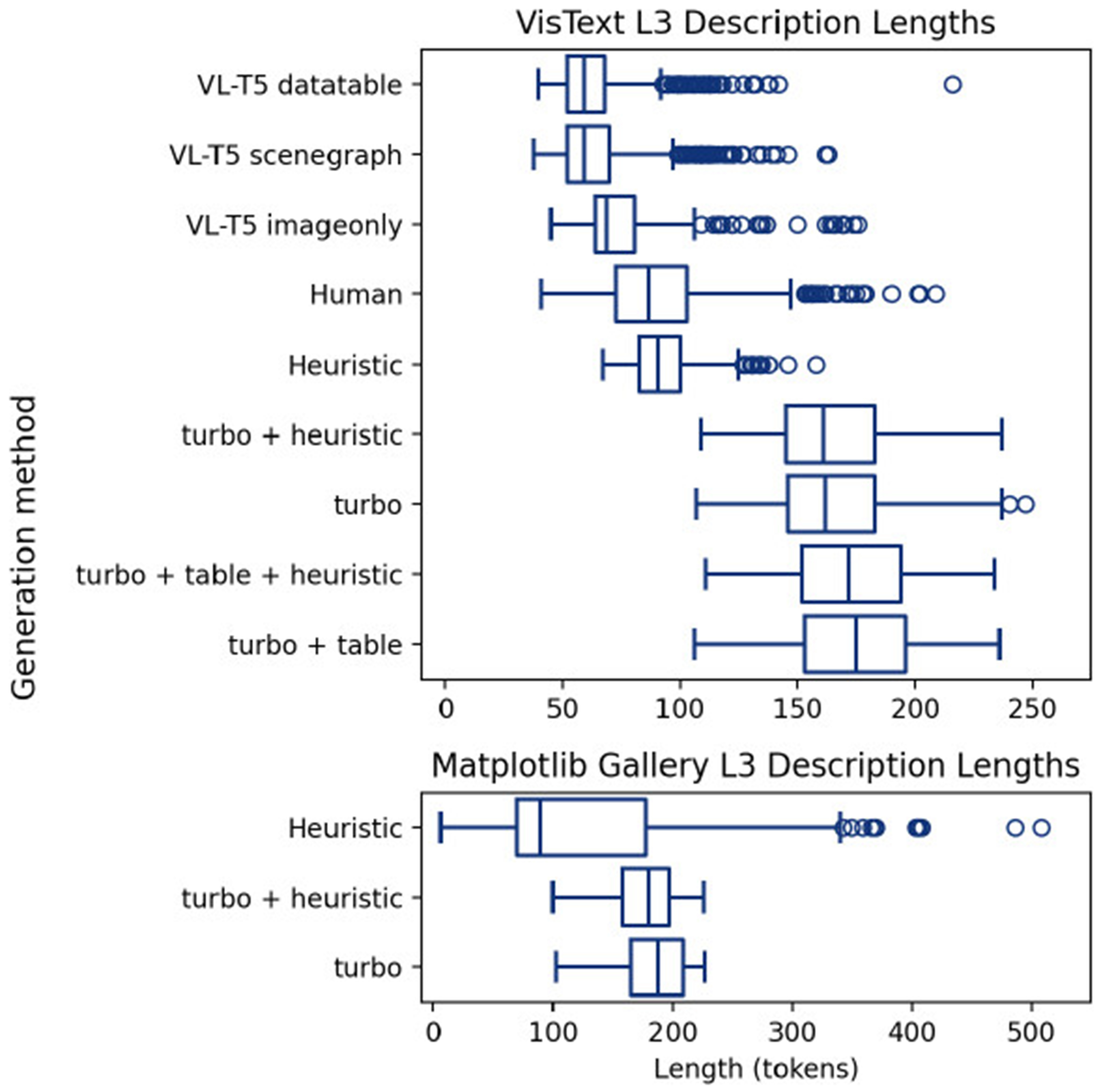
MatplotAlt is designed to generate long-form L3 descriptions for Matplotlib figures. VisText VL-T5 captions were shortest on average, followed by human, heuristic, and GPT4-turbo methods (set to a max output length of 225 tokens). Matplotlib gallery alt texts were generally longer than those for VisText figures.

Although we do not know the ideal length of VLM alt texts, prior work shows BVI users may prefer shorter captions because they find the additional information in longer descriptions more repetitive and unnecessary [HXP* 24]. In connection to this preference, we characterize the length of VLM alt texts generated with each of our methods. We measure the length of captions using nltk’s word_tokenize function [BKL09]. Figure 3 shows the distribution of alt text lengths for each generation method and dataset. On VisText, VL-T5 yielded the shortest captions with mean lengths of 62.4, 64.9, and 75.0 for image + datatable, image + scenegraph, and imageonly respectively, followed by human captions (90.0), heuristic (92.8), and the turbo models. We found that VL-T5 captions’ length was indicative of a lack of detail, with the model typically only adding one or two sentences about the overall data trend or max values. The crowdsourced captions are similarly concise, but typically contain both trends and descriptions of notable minima and maxima.

Turbo descriptions tended to use most of the token limit, and were more likely to contain additional information about outliers, specific data values, and labels. The extra context from heuristic alt text, data tables, or both increased the length of turbo alt texts even closer to the 225 token limit. For charts with simple trends, however, these longer descriptions were more prone to repetition and unnecessary detail, which are less desired by BVI users [HXP*24]. These results extend to the Matplotlib gallery dataset, with slightly longer heuristic and turbo alt text, likely due to charts with multiple subplots and more features. This difference in lengths between VisText VL-T5 / human and VLM captions indicates our shift to long-form descriptions for Matplotlib figures. The extra information in heuristic and turbo alt texts informs our fine-grained evaluation of error types in §5.4 and §5.5.

### 5.2. Similarity to Crowdsourced Captions

Similarity to human captions are widely used to measure the quality of generated alt text for data visualizations [TBS23, KLL*22]. In this section, we use two BERT-based metrics to quantify how well MatplotAlt’s L3 descriptions match the reference VisText captions.

#### 5.2.1. Caption Similarity Methods

For each generation strategy, we measure similarity to the reference human captions using Bertscore [ZKW* 19], which averages cosine similarity between the BERT [DCLT19] token embeddings for each word (ranging from −1 to 1), and BLEURT [SDP20], which uses a BERT model trained on human ratings of similarity (ranging from slightly below 0 to slightly above 1). If an image has multiple human captions, we take the average of similarities to each. A higher Bertscore indicates that the words in generated captions have meanings similar to the human captions. A cosine similarity of 1, for example, indicates that the sentences are the same, 0 indicates that they are unrelated, and −1 indicates that they have similar but opposite meanings. Similarly, a higher BLEURT indicates that the generated captions more adequately represent the overall meaning of the reference human captions. Bertscore and BLEURT together help measure how semantically similar our generated captions are to the human-written references in VisText.

Because we are not aiming to emulate the style and formatting of the VisText references, Bertscores are likely more effective than exact match n-gram similarity metrics because they capture synonyms. For example, even though “color” and “hue” are not an exact match, their sentences could still have a high Bertscore due to the high cosine similarity between their word embeddings. BLEURT is similarly less reliant on formatting, and has been shown to have slightly higher agreement with human ratings, but may also be biased towards more fluent text [SDP20]. Although n-gram overlap similarity metrics may not be appropriate due to the formatting and length differences between GPT4 and reference captions, we also measure BLEU [PRWZ02], Rouge [Lin04], and CHRF [Pop15] n-gram scores in supplemental §2 to compare with prior studies.

#### 5.2.2. Caption Similarity Results

Table 1 shows the similarity scores between Matplotalt and crowdsourced VisText descriptions for each generation strategy. We found that adding heuristic alt text in prompts to GPT4-turbo increased similarity to the human-written VisText descriptions across all metrics for both turbo + heuristic and turbo + table + heuristic, and yielded the highest Bertscore and BLEURT among our generation methods. These improvements indicate that prompting with starter alt text is a simple way to steer the style of generated captions closer to ground truth statements. Turbo + table had scores close to turbo, with small increases in Bertscore, indicating data tables do not significantly change the style of generated captions.

The finetuned VL-T5 models from VisText had higher Bertscores, but lower BLEURT. The higher Bertscore is not surprising because the VL-T5 models were trained on pairs of captions and data / images from VisText, while our generation methods are not designed to emulate the prose and length of human captions in the dataset. We find that most of these increases come from the precision component of F1 scores, while turbo descriptions have higher Bertscore recalls. This suggests that the GPT4-generated captions contain more of the semantic meaning in human references, and that their lower overall scores may be due more to the additional context and length of VLM descriptions compared to the human written captions.

### 5.3. Similarity Between Generated Descriptions and Images

Similarity scores can help quantify how well generated alt texts adhere to the style and content of human descriptions but are still dependent on the length and prose of human writers. Reference-based metrics have also been shown to be biased toward sighted user ratings of alt text over BVI user ratings [KK24]. In an attempt to evaluate Matplotalt’s descriptions independently of how well they match the dataset-specific formatting of human alt texts, we measure the similarity between caption and image embeddings.

#### 5.3.1. Multimodal Similarity Methods

We use the pretrained BLIP [LLXH22] model to measure how well our generated captions match their corresponding figures. BLIP is a multimodal encoder-decoder vision transformer model [DBK* 20] pretrained on a variety of tasks including binary classification for whether a caption matches an associated image. Using BLIP, we measure the probability that a description matches its corresponding figure, and the cosine similarity between encoded captions and figures. A high average probability suggests our captions are a good match for their corresponding Matplotlib figures, and a high cosine similarity (ranging from −1 to 1) indicates that the encoded representations of each figure and caption are aligned, suggesting that their content is similar. We average scores for all images and corresponding captions on both the VisText and Matplotlib gallery datasets. Because BLIP was not specifically trained on data visualizations, we assess its relevance for this setting in Supplemental material §1.

#### 5.3.2. Multimodal Similarity Results

Our BLIP scores on VisText were inconclusive (shown in our Supplemental material Table 3) with all matching probabilities greater than 0.9975, and all cosine similarities within 0.504 ± 0.005. Our results on the Matplotlib gallery dataset show greater variation. GPT4-turbo generations yielded the highest average matching probability (**0.9766**) and cosine similarity (**0.4879**) in this setting, followed closely by turbo + heuristic (0.9629 / 0.4717), with a larger gap to the heuristic captions (0.9270 / 0.4263). Excepting the limitations of BLIP, these scores imply that GPT4 alt texts more closely match the challenging Matplotlib gallery figures than the heuristic descriptions, and that adding heuristics in the prompt slightly reduces this alignment. At the same time, the high matching probabilities in Vis-Text and gallery datasets suggest that BLIP alone cannot measure caption *correctness*. Next, we contrast these similarity measures with a qualitative evaluation of caption errors.

### 5.4. Types of Errors in Generated Descriptions

We summarize our qualitative observations on the types of errors across generation strategies supported by MatplotAlt. We randomly sample 50 charts from the VisText test and Matplotlib gallery datasets (§4) to determine the types of errors that occur in generated descriptions. Including all of our generation methods, each VisText image had 5 descriptions, and each gallery image had 3. The lead researcher annotated a total of 400 captions. Our initial set of error types included those documented in VisText, with the researcher adding new types as they occurred in the descriptions [TBS23].

To assess relevance of these categories, two researchers on the team (including the primary) discussed the descriptions for 20 randomly selected images from each of the two sets of annotated data: adding, changing, and removing assigned error types as needed. One of the researchers involved in this process used a screen reader full time; the descriptions they annotated would serve as their ground truth in a real-world scenario. We limited this discussion to 20 images from each dataset as both researchers agreed no new error types were being discovered.

From this initial annotation, we identified 13 types of errors. Six of the error types correspond to concrete chart features in descriptions, including:

- **Value:** error in dependent variable value (e.g. wrong max value).
- **Identity:** error in independent variable (e.g. wrong max location).
- **Chart type:** wrong chart type or orientation (e.g. “line chart” instead of “scatterplot”).
- **Axis:** wrong axis range, ticks, or scale.
- **Label:** error in transcribing titles, axis labels, or other figure text.
- **Trend:** wrong direction or stability of trends (e.g. “strictly increase” vs. “generally decrease”).

In addition to these factual errors, we identify five common types of formatting mistakes:

- **Cutoff:** description ends in the middle of a sentence or claim.
- **Missing data context:** description is missing key context about L1-L3 features necessary to understand the chart. This includes failing to mention specific values for statistics and trends, even if the rest of the description is correct.
- **Unnecessary context:** Unnecessary or irrelevant L4 context is included in L3 descriptions.
- **Number name:** Failure to convert number places (e.g. “10,000 thousand” instead of “10 million”), which causes the same denomination like ‘thousand’ to be read twice by a screen reader.
- **Repetition:** The same claim is repeated multiple times.

Five of the error types (value, identity, trend, nonsense, and repetition) are also documented in VisText. We combine their direction and stability errors into a single “trend” category for simplicity.

Notably, we also distinguish between two types of model hallucinations that do not fit into the other error categories: “**nonsense**”, which are identifiable without ground truth information, and “**deceptive**”, which are undetectable without perceiving the image. While weaker language model failures are often obvious (e.g. containing disruptive grammar errors or repetition), we included this distinction because GPT4’s hallucinations overwhelmingly fit in with the rest of the description, but contain a nonexistent explanation for a trend or confidently describe a chart feature that does not exist. For example, consider the following turbo description for a chart with two line subplots: “*In the Periodogram plot, the line exhibits a jagged pattern with numerous spikes throughout the frequency range, with no single prominent peak. In contrast, the Welch plot shows a smoother line with a distinct spike around the 100 frequency marker.*” Although the smoothing explanation is correct, *both* plots have a notable spike around 100. This is particularly deceptive, suggesting the Welch method uniquely reveals a peak in the data without any other signs that the description is inaccurate.

In addition to the catchall “deceptive” error, we note that all other types except easily identifiable repetition, cutoff, grammar, or nonsense are also misleading to BVI users whose ground truth for charts may be the description.

### 5.5. Error Prevalence in Generated descriptions

In this section, we compare the frequencies of error types across each of our generation strategies and datasets.

For each dataset, we randomly select 100 figures (distinct from the 50 selected in §5.4) and manually label errors in their corresponding VL-T5 and MatplotAlt descriptions. We exclude the VL-T5 imageonly model, as we found that its captions never matched the given images. In total, we annotated 1000 figure descriptions. For each description we assign zero or more error types in order of priority based on the list in §5.4. If a caption states an incorrect value in a description of a trend but is otherwise correct, for example, we only assign it a value error, rather than value + trend or value + trend + deceptive errors. We label charts with no errors as “**correct**”, and charts without factual errors (i.e. no value, identity, axis, trend, chart type, label, deceptive, or nonsense errors) as “**value-correct**”. This means a blank caption would count as “missing context” under our criteria, but would still be value-correct.

Table 2 records the number and type of errors in the 100 captions from each dataset and generation strategy. We find that turbo + heuristic and turbo + table + heuristic produce more accurate alt texts than GPT4-turbo alone, with lower counts of value (20 → 1), identity (32 → 4), trend (22 → 14), label (34 → 3), missing context (30 → 1), and deceptive errors (22 → 6). At the same time, errors in heuristic alt text tend to carry over to turbo + heuristic and turbo + heuristic + table descriptions, even when they could be corrected with the given datatable or image. For example, almost all of the chart type errors in turbo + heuristic / turbo + table + heuristic originated with MatplotAlt’s lack of support for area charts. Excluding these chart type errors specifically on area plots, the number of correct turbo + heuristic descriptions rises to 61 and turbo + heuristic + table increases to 65. Similarly, at the time of annotation, most trend errors in turbo + heuristic captions were carried over from our simple heuristics for detecting trends.

Adding markdown data tables to prompts also increased the accuracy of descriptions, with smaller improvements in most error types, and the lowest number of trend errors out of all GPT4 generation methods. Qualitatively, we observed turbo + table and turbo + table + heuristic generations to have more detailed trend descriptions, often mentioning notable peaks and troughs outside the global min / max and periods of fuctuation not captured by other methods.

Overall, of the turbo methods, turbo + heuristic + table had the most correct captions (**50**) followed by turbo + heuristic (45), turbo + table (33), and turbo (10). This trend indicates that adding extra supervision to GPT4-turbo prompts reduces the number of deceptive factual errors in generated descriptions. Turbo’s low performance is also notable, with only 10 of its captions containing no errors, and 81/100 containing some kind of factual inaccuracy. This suggests that, out of the box, GPT4 and possibly other large VLMs have improved at generating *fluent*, but not necessarily *correct*, alt text for visualizations.

With the smaller finetuned VL-T5 models, captions tended to be more broad in addition to shorter, usually only including a line or two about general increasing and decreasing trends. We found that only 5 captions from each VL-T5 + data strategy contained mentions of specific data values, and only 3 of these from image + datatable were factually correct. This disqualified all other captions under our criteria due to missing context. 43 and 59 of the scenegraph and datatable VL-T5 captions were value-correct respectively, mostly due to omission. These results highlight the difficulty of our qualitative evaluation. In addition to not containing hallucinations, we expect heuristic and turbo alt texts to explicitly reference underlying chart data. We note that many crowdsourced L3 captions in VisText would also be labeled as missing context.

There were a lower overall number of correct captions for Matplotlib gallery figures. Our heuristic alt text in particular tended to break down in this setting due to its lack of ability to handle complex inputs, with over two thirds of its captions lacking necessary context. Mirroring our VisText results, however, heuristics still helped reduce deceptive errors in turbo + heuristic, which had the highest number of correct descriptions (39/100). This indicates that it is helpful to add heuristics in both simple univariate settings and complex ones where parsing the chart into a data table is infeasible.

Interestingly, we also labeled more of turbo’s descriptions as correct on gallery figures compared to VisText, largely due to fewer identity and trend errors. These decreases are likely because there are similarly fewer clear trends and statistics to describe in gallery figures. The content of VisText charts may have also overlapped with GPT4-turbo’s pretraining data in ways that caused identity errors. In several VisText captions, for example, turbo incorrectly labels 2008/2009 as a minimum and cites the economic crisis. This suggests that large VLMs may counter-intuitively be *less* likely to hallucinate specific data points on out-of-distribution figures, although more study is needed to confirm this claim.

## 6. Discussion

We present MatplotAlt, a Python library to facilitate adding alt text to Matplotlib figures in computational notebooks. Our design decisions to generate and surface alt text for Matplotlib figures are informed by prior work documenting barriers BVI users face in accessing visualization tools [PSTM23, SHHM23, SCWR21], and by previous systems for automatically generating alt text [MKD*23, SWM*22, LS21, SF18]. Our quantitative evaluation of the descriptions shows that MatplotAlt’s heuristic-based descriptions can adhere to the style of human-written L3 alt texts, and that prompting VLMs with heuristic descriptions can be used to steer their generations closer to ground-truth human captions without significantly harming their ability to describe complex figures. Similarly, we observe that prompting with heuristic alt text is a simple way to reduce hallucinations and improve the factuality of VLM-generated figure descriptions, even when chart data cannot be easily formatted in text. We provide recommendations to generate and surface effective image descriptions, and discuss the implications of automatically generating alt-text for BVI and sighted notebook authors. We close with limitations of our study.

### 6.1. Recommendations to generate alt text

Based on our evaluation and design considerations, we provide the following recommendations for generating alt text with MatplotAlt:

- We suggest users replace plt.show with show_with_alt or show_with_api_alt depending on context. Using heuristic alt text in domain-specific notebooks aimed at knowledgeable audiences may be desirable as they prefer concise, accurate descriptions. In more general notebooks for personal data analysis, extra VLM context may be preferred [SKZ*24].
- Our evaluation shows that prompting models with heuristic alt text and data tables increases the factuality of generations, can steer captions closer to human-written descriptions, and does not significantly harm similarity between descriptions and figures. As a result, we include heuristics and tables when possible by default in show_with_api_alt.
- Using the HTML option to surface alt text may lead to a better user experience for notebooks that are published on the web. As the accessibility of notebook interfaces is evolving, using the options to embed descriptions as code comments or markdown cell content may make these notebooks accessible to BVI users who experience barriers with notebook interfaces. Saving descriptions into image or text files, on the other hand, may make it easy to use them alongside corresponding figures in other contexts such as research publications.

### 6.2. Implications of Automating Alt Text Generation

Matplotalt provides a variety of options to generate and surface alternative texts for figures with no human input. Using these tools, BVI experts can perceive notebook visualizations that they may encounter in the wild to learn new concepts or conduct data exploration. At the same time, MatplotAlt’s current description generation methods are error-prone and could be misleading when used by BVI notebook users, as seen in our evaluation of description correctness (§5.5). We observed that the BVI research collaborator on the team was more punishing of error occurrences as he felt they mislead him on the insights he would expect to derive from the data. Future efforts could study the applicability of VLM-generated descriptions and the trust that BVI experts place in these potentially error-prone, very detailed descriptions. Additional validation support, such as access to the original data or the ability to have a sighted collaborator check results, would be helpful here. In other words, *what is the detail-accuracy tradeoff that BVI experts prefer in descriptions of data visualizations?*

To alleviate accessibility barriers posed by notebook environments, MatplotAlt provides command line tools to generate and export notebooks with described Matplotlib images. This functionality has the potential for the system to be integrated into testing and quality control processes that developers follow. For example, MatplotAlt could be added to existing git pipelines that authors use to test notebooks.

### 6.3. Implications for interactive data exploration

Several efforts equip BVI users with the tools to interactively explore data [PTD*22, TMS*23]. MatplotAlt is complimentary to these; it provides a standardized mode to access alternative text and requires little to no change in notebook hosting environments. Future efforts could add question-answering abilities to MatplotAlt, making the data exploration process for BVI users iterable. Similarly, MatplotAlt’s generation mechanisms could be integrated into existing data exploration tools such as MAIDR and Umwelt [SXL*24, SKZ*24, ZPPC*24]. The approaches explored in MatplotAlt to use underlying data to enhance descriptions could inform future efforts to create interactive sonifications to help BVI users answer their questions.

### 6.4. Limitations

MatplotAlt supports multiple ways of generating and surfacing alt text for Matplotlib figures. Alternative text and figure descriptions alone, however, do not support the full extent of data exploration needs of BVI experts [SCWR21,SWM*22]. We believe that alternative text and text descriptions, however, serve as a standardized way of exposing accessible graphics across a variety of scenarios and serve as an important first step. Future versions of MatplotAlt could offer functionality for developers to interact with the data via Q and A to support interactive explorations of data by integrating with systems like MAIDR [SKZ*24].

Our evaluations of alt text generations may not be perceived as rigorous as they do not offer definitive guidelines of specific generation strategies of MatplotAlt as good defaults. Given the variability in alternative text quality across different measures as shown in our evaluations, and varying preferences of BVI users on alt text [SKZ*24], providing definitive defaults that work for all contexts is not feasible. We trust the developers producing notebooks with Matplotlib figures to use their best judgment and expertise to decide on a good alt-text generation strategy. Although the desc_level parameter allows users to modify the inclusion of information, we leave it to feature work to support changing the verbosity and ordering of generated descriptions, which are also important modes of customization for BVI users [JPPH*24].

Additionally, our generation strategies may not be effective for several chart types or plotting methods used by Matplotlib users as our evaluations used carefully curated images. Future work could programmatically evaluate our methods on a larger subset of the Jetbrains dataset [Guz], which may be more representative of MatplotAlt use in-the-wild. The alttextify command begins to make the infrastructure to run such at-scale evaluations available, but still requires executing arbitrary code, which is potentially dangerous in untrusted notebooks.

### 6.5. Conclusion

We present MatplotAlt, a Python library to add alt text to Matplotlib figures in computational notebooks. MatplotAlt implements functions to generate and embed or export figure descriptions in a single line of code or command. Our initial qualitative evaluations indicate that MatplotAlt can generate effective alt text for Matplotlib figures, and that heuristic alt text and data tables can be used to increase the factuality of VLM descriptions. Our goal is that this work will eventually see adoption and start increasing the percentage of notebooks on the web that are accessible to BVI users. We also hope that our project will gain collaborators (including from the Matplotlib and Jupyter teams) who contribute their experience to improve its accessibility and generations.

## Supplementary Material

Supplementary material

## Figures and Tables

**Figure 1:**
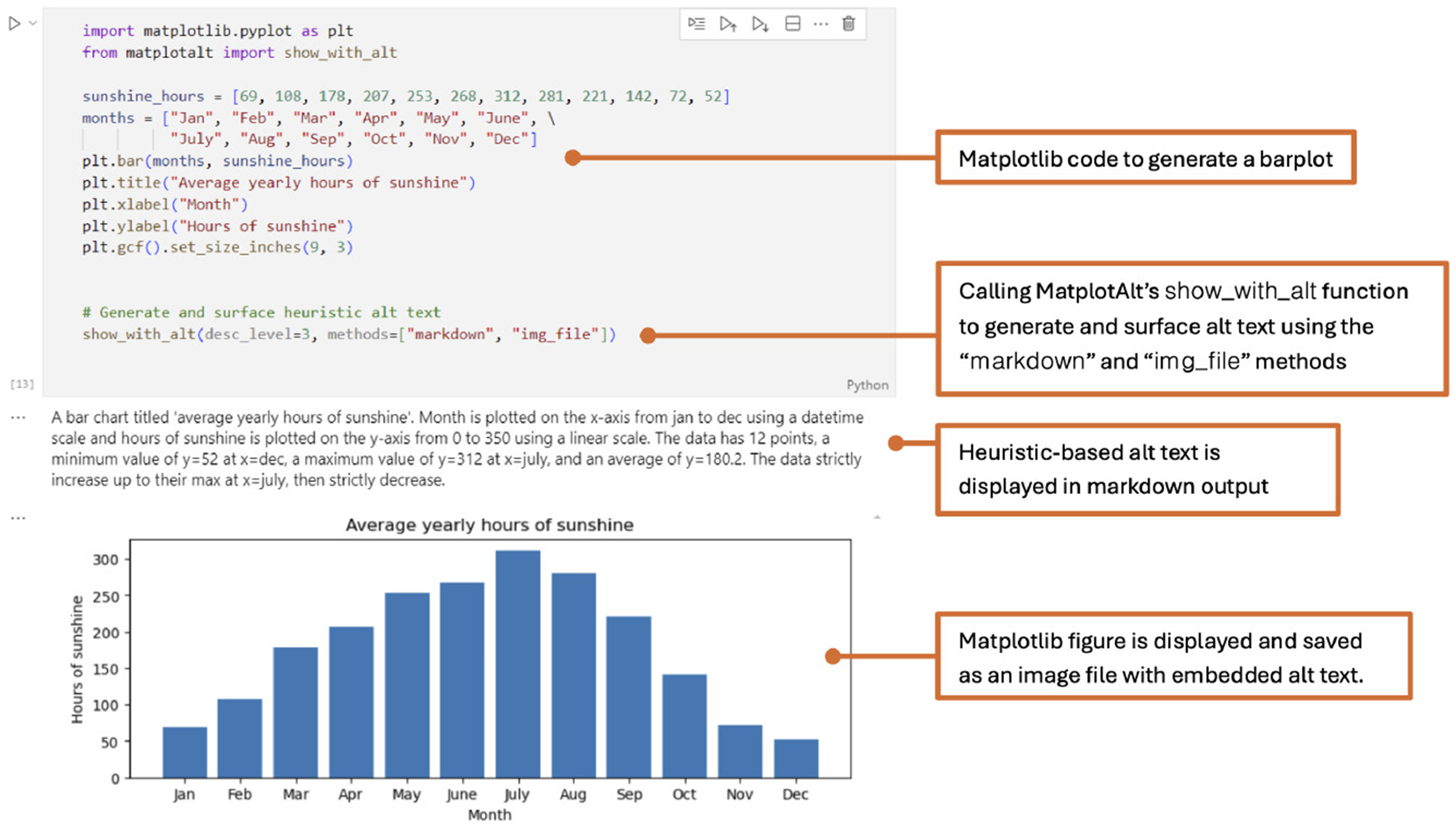
MatplotAlt can be used to generate and surface Matplotlib alt text in a single line of code. In this example, we call MatplotAlt’s show_with_alt function after creating a Matplotlib bar chart to display heuristic-based alt text in markdown and embedded in a saved version of the figure. desc_level=3 indicates that the description includes encodings, statistics, and trends. MatplotAlt also provides options to embed alt text directly in Jupyter figures, and generate descriptions using vision language models.

**Table 1:** Adding heuristic alt text to GPT4-prompts can steer generations closer to human-written figure captions. Similarity between reference and generated descriptions for each method. Higher (blue) is better for each metric. VisText VL-T5 models produce text with the highest BertScore precision and F1, while turbo + heuristic generations typically have the highest Bertscore recall and BLEURT.

VisText Alt Text Type	BertScore F1	BertScore recall	BertScore precision	BLEURT
VL-T5 image only	0.8613	0.8546	0.8685	−1.042
VL-T5 image + scenegraph	**0.9150**	0.8939	**0.9375**	−0.2919
VL-T5 image + table	**0.9161**	0.8949	**0.9386**	−0.2653

Heuristic	0.8850	0.8953	0.8753	−0.1526
turbo	0.8826	0.8954	0.8705	−0.1439
turbo + table	0.8825	0.8974	0.8683	−0.1370
turbo + heuristic	0.8875	**0.9022**	0.8736	**−0.0944**
turbo + table + heuristic	0.8865	**0.9024**	0.8713	**−0.1000**

**Table 2:** MatplotAlt’s heuristic and VLM methods can produce factually correct and informative long-form alt text, but VLMs still struggle without extra supervision about chart data and features. For 100 descriptions from each generation strategy and dataset, this table shows the number labeled as correct and value-correct (darker blue is better), and the number containing each type of error (darker red is worse).

Dataset	Alt Text Type	Correct	Value-Correct	Chart Type	Axis	Value	Identity	Trend	Label	Missing Ctx.	Unnec. Ctx.	Repetition	Cutoff	Number Name	Deceptive	Nonsense
VisText	VL-T5 - image + scene graph	0	43	**0**	**1**	4	30	**24**	**1**	95	**1**	6	**0**	**1**	**0**	13
VisText	VL-T5 - image + table	**3**	**59**	**0**	**1**	**1**	**12**	**24**	**1**	95	**1**	**0**	**0**	**0**	3	3

VisText	Heuristic	**48**	**57**	30	**0**	**0**	6	16	**0**	11	**0**	11	**0**	**0**	**0**	**0**
VisText	turbo	10	19	**15**	10	20	32	22	34	30	**1**	**0**	4	3	22	**0**
VisText	turbo + heuristic	45	54	28	5	**1**	**4**	14	3	**1**	4	**2**	**1**	7	6	**0**
VisText	turbo + table	33	53	**17**	11	4	10	8	21	10	**1**	**2**	8	5	15	5
VisText	turbo + heuristic + table	**50**	**57**	26	5	**0**	**2**	11	**0**	**1**	3	**0**	7	5	5	3

Gallery	Heuristic	10	**65**	6	**9**	**8**	20	**4**	**0**	67	**0**	25	**1**	**0**	**1**	**1**
Gallery	turbo	33	46	9	**10**	**7**	**9**	**3**	4	6	5	**0**	20	**0**	31	**0**
Gallery	turbo + heuristic	**39**	59	**1**	**9**	**6**	20	**4**	**1**	**3**	4	**0**	34	**0**	14	**2**
