# Supplementary material for: MatplotAlt: A Python Library for Adding Alt Text to Matplotlib Figures in Computational Notebooks
Source: Comput Graph Forum. Author manuscript; Available in PMC 2026 Mar 4. (PMC12955807; doi:10.1111/cgf.70119)
Supplement: Supplementary material [file NIHMS2114346-supplement-Supplementary_material.pdf]

1. Example Gallery

We provide a gallery of MatplotAlt's supported chart types in Table 1, a list of their corresponding heuristic-based alt texts in Table 2, and an example data table created using `generate_alt_text` in Table 3. We also compare each of our alt text generation methods on two charts in Tables 4 and 5, with sections labeled by L1-L3 semantic level. Finally, we provide two examples from VisText and the Matplotlib gallery, labeled with a range of different error types in Table 6.

2. Heuristic Alt Text Templates

While our heuristic alt text is formatted differently from figure to figure, we use the following templates in most cases. Our L1 description is usually:

```
a [chart type] titled [title].
[x-label] is plotted on the x-axis
from [x-axis min] to [x-axis max]
using a [x-axis scale] scale.
[y-label] is plotted on the y-axis
from [y-axis min] to [y-axis max],
...
```

For each variable encoded with a color we add:

```
[variable label] is plotted in [label
color encoding].
```

And for each annotation we add:

```
An annotation at [annotation x/y
position] reads [annotation text].
```

For L2 descriptions, we compute relevant statistics from the data depending on the chart type and then describe them as:

```
[variable label] has a [statistic
name] of [dependent axis]=[value]
at [independent axis]=[value], a
[statistic name] of....
```

Several statistics do not fit this format. For example: `The max contour is centered around [x, y].`

For plots displaying one or two-dimensional data, we generate simple trend summaries from the difference between subsequent points. For instance:

```
[variable name] strictly increase to
a max at [dependent axis]=[end point],
then generally decrease.
```

Future implementations may use more involved methods to capture complex trends like fitting piecewise functions. To see a range of the different outputs possible with `generate_alt_text`, we provide more examples at `https://github.com/make4all/matplotalt/blob/main/examples/examples.ipynb`

3. Vision Language Model Prompts

We use different versions of our instruction prompt based on the given description level and other config options.

L1:

```
You are a helpful assistant that describes
figures. Here are two example descriptions:
1. 'This is a vertical bar chart entitled
'COVID-19 mortality rate by age' that plots
Mortality rate by Age. Mortality rate is
plotted on the vertical y-axis from 0 to
15%. Age is plotted on the horizontal x-axis
in bins: 10-19, 20-29, 30-39, 40-49, 50-59,
60-69, 70-79, 80+.' 2. 'This is a line chart
titled 'Big Tech Stock Prices' that plots
price by date. The corporations include AAPL
(Apple), AMZN (Amazon), GOOG (Google), IBM
(IBM), and MSFT (Microsoft). The years are
plotted on the horizontal x-axis from 2000
to 2010 with an increment of 2 years. The
prices are plotted on the vertical y-axis
from 0 to 800 with an increment of 200.'

 Only include information about the chart
type, title, axis ranges, and labels.
Be concise and limit your response to
{max_tokens} tokens.
```

L2:

```
You are a helpful assistant that describes
figures. Here are two example descriptions:
1. 'This is a vertical bar chart entitled
'COVID-19 mortality rate by age' that plots
Mortality rate by Age. Mortality rate is
plotted on the vertical y-axis from 0
to 15%. Age is plotted on the horizontal
x-axis in bins: 10-19, 20-29, 30-39, 40-49,
50-59, 60-69, 70-79, 80+. The highest
COVID-19 mortality rate is in the 80+ age
range, while the lowest mortality rate is
in 10-19, 20-29, 30-39, sharing the same
rate. COVID-19 mortality rate does not
linearly correspond to the demographic age.'
2. 'This is a line chart titled 'Big Tech
Stock Prices' that plots price by date. The
corporations include AAPL (Apple), AMZN
(Amazon), GOOG (Google), IBM (IBM), and MSFT
(Microsoft). The years are plotted on the
horizontal x-axis from 2000 to 2010 with an
increment of 2 years. The prices are plotted
on the vertical y-axis from 0 to 800 with
an increment of 200. GOOG has the greatest
price over time. MSFT has the lowest price
over time.'

 Include information about the chart type,
title, axis ranges, and labels. If possible,
describe statistics, extrema, outliers,
correlations, and point-wise comparisons
```

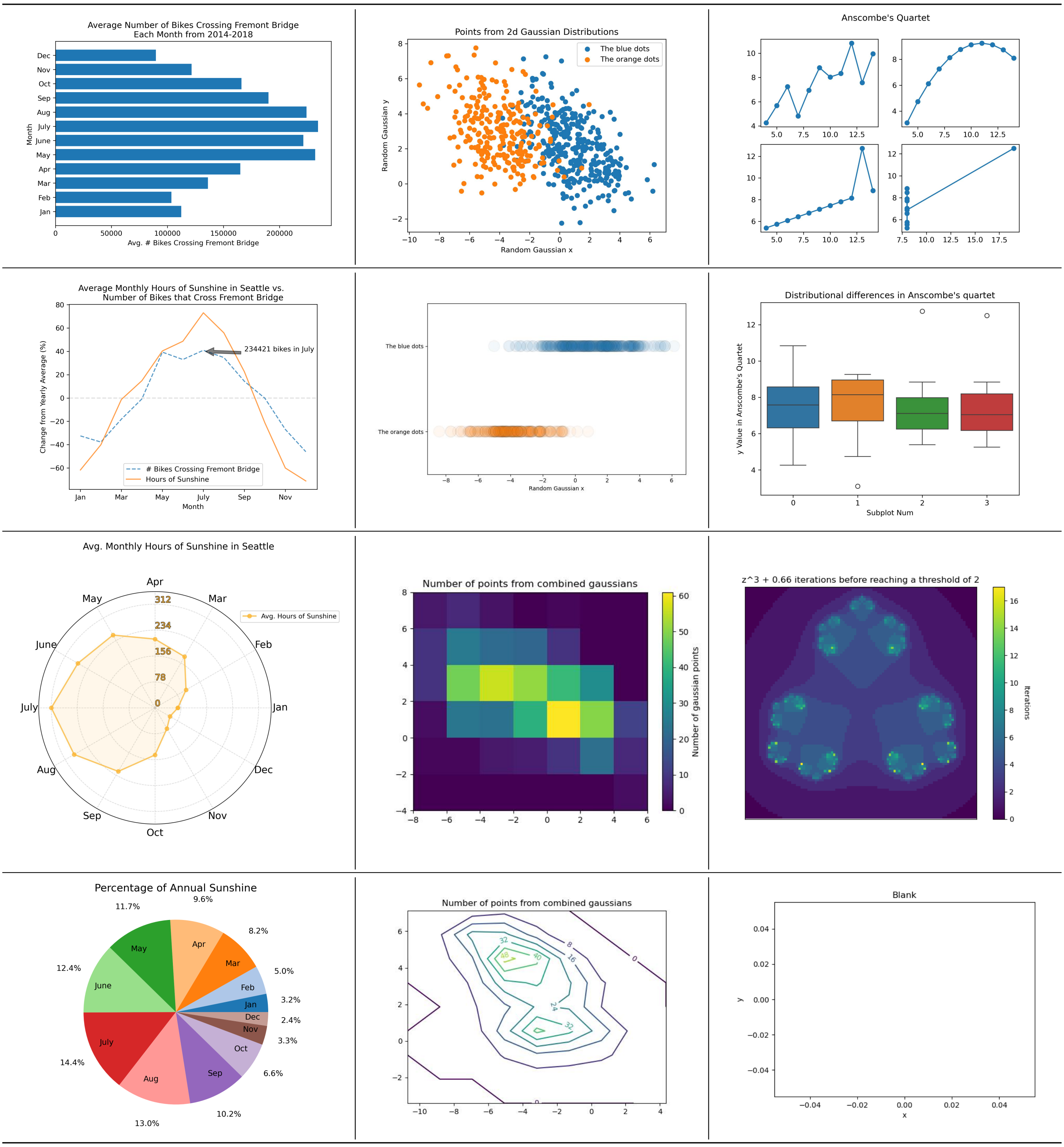

Table 1: *Types of charts supported by MatplotAlt. From top left to bottom right: a bar plot, a scatter plot, multiple subplots, a line plot, a strip plot, a boxplot, a radial line plot, a heatmap, an image displayed with* `imshow`*, a pie chart, a contour plot, and a blank plot. Example heuristic alt texts for each of the charts are listed in Table 2.*

Plot type	Example Heuristic-based Alt Text
**Bar plot**	A bar chart titled 'average number of bikes crossing fremont bridge each month from 2014-2018'. Avg. bikes crossing fremont bridge is plotted on the x-axis from 0 to 250000 using a linear scale and month is plotted on the y-axis from jan to dec using a datetime scale. The data has 12 points, a minimum value of x=89700 at y=dec, a maximum value of x=234400 at y=july, and an average of x=166300.
**Line plot**	A line plot titled 'average monthly hours of sunshine in seattle vs. Number of bikes that cross fremont bridge'. Month is plotted on the x-axis from jan to nov using a datetime scale and change from yearly average (%) is plotted on the y-axis from -80 to 100 using a linear scale. bikes crossing fremont bridge is plotted in dark blue and hours of sunshine is plotted in orange. There is a horizontal line at y=0.0. An annotation reads '234421 bikes in july'. bikes crossing fremont bridge has a minimum value of y=-46.08 at x=12, a maximum value of y=40.93 at x=7, and an average of y=0. Hours of sunshine has a minimum value of y=-71.15 at x=12, a maximum value of y=73.09 at x=7, and an average of y=0.
**Pie chart**	A pie chart titled 'percentage of annual sunshine'. There are 12 slices: jan (3.19%), feb (4.993%), mar (8.229%), apr (9.57%), may (11.7%), june (12.39%), july (14.42%), aug (12.99%), sep (10.22%), oct (6.565%), nov (3.329%), and dec (2.404%). The data has a standard deviation of x=4.006, an average of x=8.333, a maximum value of x=14.42, and a minimum value of x=2.404. The data strictly increase up to their max at x=14.42, then strictly decrease.
**Radial line chart**	A radial line plot titled 'avg. Monthly hours of sunshine in seattle'. The x-axis ranges from jan to dec using a datetime scale and the y-axis ranges from 0 to 312 using a linear scale. Avg. Hours of sunshine is plotted in orange. Avg. Hours of sunshine has a minimum value of y=52 at x=dec, a maximum value of y=312 at x=july, and an average of y=180.2.
**Scatter plot**	A scatter plot titled 'points from 2d gaussian distributions'. Random gaussian x is plotted on the x-axis from -12.5 to 7.5 and random gaussian y is plotted on the y-axis from -4 to 10, both using linear scales. The blue dots is plotted in dark blue and the orange dots is plotted in orange. The blue dots has 350 points, an average of x=1.149, an average of y=1.915, a linear fit of y=-0.5268x+2.52, and 6 outliers. The orange dots has 250 points, an average of x=-4.114, an average of y=3.055, a linear fit of y=-0.3893x+1.453, and 5 outliers.
**Strip plot**	A strip plot. Random gaussian x is plotted on the x-axis from -10 to 8 using a linear scale and the y-axis ranges from the blue dots to the orange dots using a categorical scale. Strip 1 has 350 points, a median of x=0.9834, and 1 outlier at x=-5.01. Strip 2 has 250 points, a median of x=-3.955, and 3 outliers.
**Heatmap**	A 7x6 heatmap titled 'number of points from combined gaussians'. The x-axis ranges from -8 to 6, the y-axis ranges from -4 to 8, and number of gaussian points is plotted on the z-axis from 0 to 61, all using linear scales. The data has a minimum value of z=0 at (-8, -4), a maximum value of z=61 at (0, -4), and an average of z=14.19.
**Contour plot**	A contour plot titled 'number of points from combined gaussians'. The x-axis ranges from -12 to 6 and the y-axis ranges from -4 to 8, both using linear scales. 9 contour lines are plotted with values 0, 0, 8, 16, 24, 32, 32, 40, and 48. The max contour is centered around (-4.803, 4.432).
**Multiple subplots**	A figure with 4 subplots titled 'anscombe's quartet'. *subplot 1*: a line plot. The x-axis ranges from 2.5 to 15 and the y-axis ranges from 2 to 12, both using linear scales. The data are plotted in dark blue. The data has a linear fit of y=0.5001x+3, an average of y=7.501, and a standard deviation of y=1.937. The data generally increase up to their max at x=12. *subplot 2*: a line plot. The x-axis ranges from 2.5 to 15 and the y-axis ranges from 2 to 10, both using linear scales. The data are plotted in dark blue. The data has a linear fit of y=0.5x+3.001, an average of y=7.501, and a standard deviation of y=1.937. The data strictly increase up to their max at x=11, then strictly decrease. *subplot 3*: a line plot. The x-axis ranges from 2.5 to 15 and the y-axis ranges from 4 to 14, both using linear scales. The data are plotted in dark blue. The data has a linear fit of y=0.4997x+3.002, an average of y=7.5, and a standard deviation of y=1.936. The data strictly increase up to their max at x=13, then strictly decrease. *subplot 4*: a line plot. The x-axis ranges from 5 to 20 and the y-axis ranges from 4 to 14, both using linear scales. The data are plotted in dark blue. The data has a linear fit of y=0.4999x+3.002, an average of y=7.501, and a standard deviation of y=1.936.
**Boxplot**	A boxplot titled 'distributional differences in anscombe's quartet'. Subplot num is plotted on the x-axis from 0 to 3 and y value in anscombe's quartet is plotted on the y-axis from 2 to 14, both using linear scales. Boxplot 0 has a median of 7.58, an interquartile range of 2.255, and no outliers. Boxplot 1 has a median of 8.14, an interquartile range of 2.255, and 1 outlier at y=3.1. Boxplot 2 has a median of 7.11, an interquartile range of 1.73, and 1 outlier at y=12.74. Boxplot 3 has a median of 7.04, an interquartile range of 2.02, and 1 outlier at y=12.5.
**Image**	A 50x50 image titled 'z$\hat{3}$ + 0.66 iterations before reaching a threshold of 2'. The x-axis ranges from -0.5 to 49.5 using a numerical scale, the y-axis ranges from -0.5 to 49.5 using a linear scale, and iterations is plotted on the z-axis from 0 to 17 using a linear scale. The data has a minimum value of z=0 at (0, 0), a maximum value of z=17 at (38, 12), and an average of z=2.511.
**Blank plot**	A blank plot titled 'blank'. X is plotted on the x-axis from -0.06 to 0.06 and y is plotted on the y-axis from -0.06 to 0.06, both using linear scales.

Table 2: *Heuristic figure descriptions for each of the gallery figures created by calling* `show_with_alt`

month	# bikes crossing fremont bridge (change from yearly average (%))	hours of sunshine (change from yearly average (%))
0	-32.52	-61.72
1	-37.78	-40.08
2	-18.13	-1.248
3	-0.7941	14.84
4	39.35	40.36
5	33.02	48.68
6	40.93	73.09
7	34.72	55.89
8	14.37	22.61
9	-0.1582	-21.22
10	-26.93	-60.06
11	-46.08	-71.15

Table 3: *An example markdown table created using* `generate_alt_text` *(converted to LaTeX formatting)*

between variables. Be concise and limit your response to {max_tokens} tokens.

L3:

You are a helpful assistant that describes figures. Here are two example descriptions: 1. 'This is a vertical bar chart entitled 'COVID-19 mortality rate by age' that plots Mortality rate by Age. Mortality rate is plotted on the vertical y-axis from 0 to 15%. Age is plotted on the horizontal x-axis in bins: 10-19, 20-29, 30-39, 40-49, 50-59, 60-69, 70-79, 80+. The highest COVID-19 mortality rate is in the 80+ age range, while the lowest mortality rate is in 10-19, 20-29, 30-39, sharing the same rate. COVID-19 mortality rate does not linearly correspond to the demographic age. The mortality rate increases with age, especially around 40-49 years and upwards. The mortality rate increases exponentially with older people.' 2. 'This is a line chart titled 'Big Tech Stock Prices' that plots price by date. The corporations include AAPL (Apple), AMZN (Amazon), GOOG (Google), IBM (IBM), and MSFT (Microsoft). The years are plotted on the horizontal x-axis from 2000 to 2010 with an increment of 2 years. The prices are plotted on the vertical y-axis from 0 to 800 with an increment of 200. GOOG has the greatest price over time. MSFT has the lowest price over time. Prices of particular Big Tech corporations seem to fluctuate but nevertheless increase over time. Years 2008-2009 are exceptions as we can see an extreme drop in prices of all given corporations.'

Include information about the chart type, title, axis ranges, and labels. If possible, describe statistics, extrema, outliers, correlations, point-wise comparisons, and trends for each plotted variable. Be concise and limit your response to {max_tokens} tokens.

L4:

You are a helpful assistant that describes figures. Here are two example descriptions: 1. 'This is a vertical bar chart entitled 'COVID-19 mortality rate by age' that plots Mortality rate by Age. Mortality rate is plotted on the vertical y-axis from 0 to 15%. Age is plotted on the horizontal x-axis in bins: 10-19, 20-29, 30-39, 40-49, 50-59, 60-69, 70-79, 80+. The highest COVID-19 mortality rate is in the 80+ age range, while the lowest mortality rate is in 10-19, 20-29, 30-39, sharing the same rate. COVID-19 mortality rate does not linearly correspond to the demographic age. The mortality rate increases with age, especially around 40-49 years and upwards. This relates to people's decrease in their immunity and the increase of co-morbidity with age. The mortality rate increases exponentially with older people.' 2. 'This is a line chart titled 'Big Tech Stock Prices' that plots price by date. The corporations include AAPL (Apple), AMZN (Amazon), GOOG (Google), IBM (IBM), and MSFT (Microsoft). The years are plotted on the horizontal x-axis from 2000 to 2010 with an increment of 2 years. The prices are plotted on the vertical y-axis from 0 to 800 with an increment of 200. GOOG has the greatest price over time. MSFT has the lowest price over time. Prices of particular Big Tech corporations seem to

Alt Text Type	Sunshine and Bikes Example Alt Text	Anscombe's Quartet Example Alt Text
Figure Image	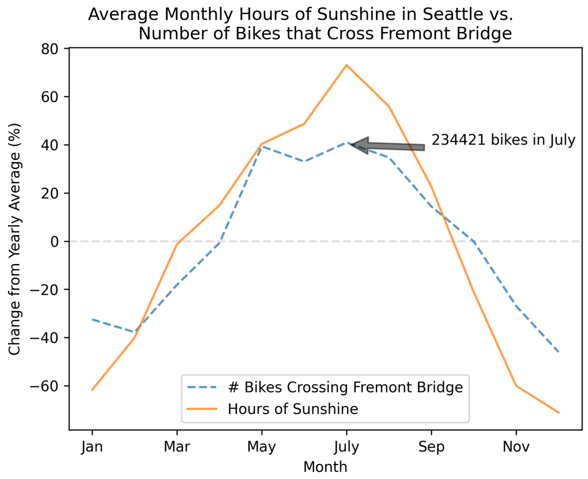	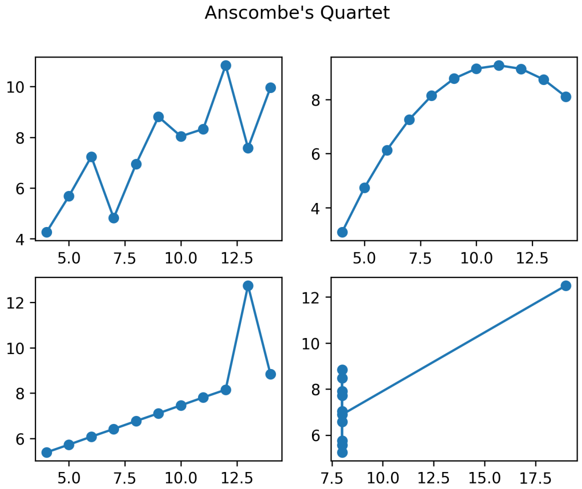
Heuristic	[L1] A line plot titled 'average monthly hours of sunshine in seattle vs. Number of bikes that cross fremont bridge'. Month is plotted on the x-axis from jan to nov using a datetime scale and change from yearly average (%) is plotted on the y-axis from -80 to 100 using a linear scale. # bikes crossing fremont bridge is plotted in dark blue and hours of sunshine is plotted in orange. There is a horizontal line at y=0.0. An annotation reads '234421 bikes in july'. [L2] # bikes crossing fremont bridge has a minimum value of y= 46.08 at x=12, a maximum value of y=40.93 at x=7, and an average of y=0. Hours of sunshine has a minimum value of y= 71.15 at x=12, a maximum value of y=73.09 at x=7, and an average of y=0. [L3] Hours of sunshine strictly increase up to their max at x=7, then strictly decrease. [L2] # bikes crossing fremont bridge and hours of sunshine have a correlation of 0.95.	[L1] A figure with 4 subplots titled 'anscombe's quartet'.<br>**subplot 1**: a line plot. The x-axis ranges from 2.5 to 15 and the y-axis ranges from 2 to 12, both using linear scales. The data are plotted in dark blue. [L2] The data has a minimum value of y=4.26 at x=4, a maximum value of y=10.84 at x=12, and an average of y=7.501. [L3] The data generally increase up to their max at x=12.<br>[L1] **subplot 2**: a line plot. The x-axis ranges from 2.5 to 15 and the y-axis ranges from 2 to 10, both using linear scales. The data are plotted in dark blue. [L2] The data has a minimum value of y=3.1 at x=4, a maximum value of y=9.26 at x=11, and an average of y=7.501. [L3] The data strictly increase up to their max at x=11, then strictly decrease.<br>[L1] **subplot 3**: a line plot. The x-axis ranges from 2.5 to 15 and the y-axis ranges from 4 to 14, both using linear scales. The data are plotted in dark blue. [L2] The data has a minimum value of y=5.39 at x=4, a maximum value of y=12.74 at x=13, and an average of y=7.5. [L3] The data strictly increase up to their max at x=13, then strictly decrease.<br>[L1] **subplot 4**: a line plot. The x-axis ranges from 5 to 20 and the y-axis ranges from 4 to 14, both using linear scales. The data are plotted in dark blue. [L2] The data has a minimum value of y=5.25 at x=8, a maximum value of y=12.5 at x=19, and an average of y=7.501.
turbo	[L1] This is a line chart titled 'Average Monthly Hours of Sunshine in Seattle vs. Number of Bikes that Cross Fremont Bridge'. Two variables are plotted: the number of bikes crossing Fremont Bridge is shown with a dashed blue line, and the hours of sunshine with a solid orange line. Both variables are compared against the months of the year, plotted on the horizontal x axis from January to November. The vertical y axis represents the change from yearly average, marked in percentages ranging from 60% to 80%. [L2] The number of bikes peaks in July at 234421 bikes, which correlates with the highest point of the hours of sunshine. [L3] The winter months show a decrease in both bikes crossing and sunshine hours. [L4] This suggests a seasonal pattern where more people bike during sunnier months. [L2] The chart demonstrates a clear positive correlation between the number of bikers and the amount of sunshine.	[L1] This figure displays Anscombe's Quartet, consisting of four scatter plots, each depicting a different dataset. The x-axis ranges from about 4 to 20, and the y axis ranges from roughly 3 to 14 across the four charts. [L2] All datasets exhibit a similar statistical summary yet present distinct distributions:<br>[L3] **Top-left plot**: Appears to show a linear relationship with some variability.<br>**Top-right plot**: Displays a curved, quadratic-like relationship.<br>**Bottom-left plot**: Mostly linear but with a clear outlier that deviates significantly from the trend.<br>**Bottom-right plot**: Shows a strong linear relationship with an outlier that lies vertically far from the line.<br>[L1] Each plot's data points are in blue with connecting lines. [L4] The consistency of these statistical properties, despite differing datasets, underscores the importance of graphically analyzing data to avoid misleading interpretations based only on statistical summaries.
turbo + heuristic	[L1] This line chart depicts 'Average Monthly Hours of Sunshine in Seattle vs. Number of Bikes that Cross Fremont Bridge'. The x-axis represents months, ranging from January to November, while the y-axis shows the percentage change from the yearly average, ranging from -80% to +100%. Two data series are plotted: the number of bikes crossing Fremont Bridge is illustrated with a dark blue dashed line, and the hours of sunshine are shown with a solid orange line. A horizontal reference line is drawn at y=0%.<br>[L2] The data shows that both variables reach their peak in July, with the hours of sunshine increasing to a maximum of 73.09% and the number of bikes reaching a peak change of 40.93%, marked by an annotation indicating "234421 bikes in July". Conversely, December experiences the lowest points for both variables, with bikes at -46.08% and sunshine at -71.15%. The overall trend suggests a strong correlation (0.95) between sunshine and cycling activity, [L4] reflecting a potential increase in outdoor activities during sunnier months.	[L1] The figure consists of four subplots collectively titled "Anscombe's Quartet," each displaying a line plot with data points in dark blue.<br>**Subplot 1**: x axis 2.5 15, y axis 2 12. [L2/L3] Data rises to y=10.84 at x=12 then falls slightly.<br>**Subplot 2**: [L1] x-axis 2.5-15, y-axis 2-10. [L2/L3] Data climbs to y=9.26 at x=11 then decreases.<br>**Subplot 3**: [L1] x-axis 2.5-15, y-axis 4-14. [L2/L3] Data ascends to y=12.74 at x=13, followed by a decline.<br>**Subplot 4**: [L1] x-axis 5-20, y-axis 4-14. [L2/L3] Data shows a strong linear relationship from the lower left to the upper right corner.<br>[L2] Each subplot shares a mean of y=7.5 (approx.) [L4] but displays distinct patterns, illustrating how identical statistical properties can arise from very different data distributions.

Table 4: *MatplotAlt example alt text for two figures using each generation strategy. Continued in Table 5 for turbo + table and turbo + table + heuristic methods. Text is color-coded and tagged by semantic level. Emphasis Added for readability.*

Alt Text Type	Sunshine and Bikes Example Alt Text	Anscombe's Quartet Example Alt Text
turbo + heuristic + table	[L1]This is a line chart titled 'Average Monthly Hours of Sunshine in Seattle vs. Number of Bikes that Cross Fremont Bridge'. It compares the monthly change from the yearly average for the number of bikes crossing Fremont Bridge (dashed line) and the hours of sunshine (solid line) in Seattle. The horizontal x-axis labels the months from January to December, while the vertical y-axis represents the percent change from the yearly average, ranging from -80% to 60%.<br><br>[L3] Both variables show seasonality with values rising in the warmer months and falling in the cooler months. [L2] The highest point is in July for both variables, with hours of sunshine around 73% above the average and a peak in bike crossings at 234421, which corresponds to approximately a 41% increase. The chart suggests a positive correlation between the amount of sunshine and the number of bicycles crossing the bridge.	[L1] This figure presents Anscombe's Quartet, comprising four subplots of line plots, each depicting a different trend. The title "Anscombe's Quartet" is located at the top. Each plot has its data points marked in dark blue, [L3] with the plots varying in data distribution and trends.<br>[L1] **Subplot 1**: The x axis ranges from 4 to 14, and the y axis ranges from 2 to 12. [L3] The data display a somewhat erratic pattern, with fluctuations but generally trending upwards. [L2] A peak is observed at y=10.84 with x=12.<br>[L1] **Subplot 2**: The x axis is from 4 to 14, and the y axis from 2 to 10. [L2 / L3] The data exhibit a highly curved trend, first rising to a peak of y=9.26 at x=11 then declining smoothly.<br>[L1] **Subplot 3**: The x axis is between 4 to 14, and the y axis extends from 4 to 14. [L2 / L3] The line plot shows a continuous increase up to a sharp peak at y=12.74, x=13, followed by a drop.<br>[L1] **Subplot 4**: Here, the x axis ranges from 8 to 19, while the y axis spans from 4 to 14. [L3] The data is tightly clustered at x=8, except for an outlier at x=19
turbo + heuristic + table	[L1] This is a line chart titled 'Average Monthly Hours of Sunshine in Seattle vs. Number of Bikes That Cross Fremont Bridge'. The months are plotted from January to November on the horizontal x axis and the change from yearly average is denoted in percentage on the vertical y axis ranging from 80% to 100%. The number of bikes crossing Fremont Bridge is represented by a dashed line in dark blue, while hours of sunshine is illustrated with a solid orange line. [L2] There is a strong positive correlation between the two variables, with a correlation coefficient of 0.95. Both metrics peak in July, where the number of bikes reaches a 40.93% increase from the yearly average, with 234,421 bikes recorded in that month. Simultaneously, hours of sunshine cap at a 73.09% increase. [L3] Both variables exhibit similar seasonal patterns, with higher values in the warmer months and lower values in the colder months, [L4] reflecting the influence of weather on outdoor activities such as biking.	[L1] This figure, titled 'Anscombe's Quartet', consists of four subplots with line plots of dark blue data points. [L4] Each chart has a distinct pattern, despite having the same mean and variance, demonstrating different data distributions that yield similar descriptive statistics.<br>[L1] **Subplot 1**'s x axis ranges from 2.5 to 15, and y axis from 2 to 12. [L2/L3] Data points generally increase, reaching a maximum at x=12, y=10.84.<br>[L1] **Subplot 2**'s x- and y axes share the same range as the first, [L2/L3] but the data strictly increase to a maximum at x=11, y=9.26, then strictly decrease.<br>[L1] **Subplot 3**'s x-axis is the same, with the y axis ranging from 4 to 14. [L2/L3] Data strictly increase, peaking at x=13, y=12.74, before decreasing.<br>[L1] **Subplot 4** features an x-axis from 5 to 20 and a y axis from 4 to 14. [L2/L3] Most data cluster at x=8, with an outlier at x=19, y=12.5, creating a steep incline.<br>[L4] This quartet is commonly used to illustrate the importance of graphing data before analyzing it and the limitations of summary statistics.

Table 5: *MatplotAlt example alt text for two figures using each generation strategy. Continued in Table 4 for Heuristic, turbo, and turbo + heuristic methods. Text is color-coded and tagged by semantic level. Emphasis Added for readability.*

`fluctuate but nevertheless increase over time. Years 2008-2009 are exceptions as we can see an extreme drop in prices of all given corporations. The big drop in prices was caused by financial crisis of 2007-2008. The crisis culminated with the bankruptcy of Lehman Brothers on September 15, 2008 and an international banking crisis.'`

`Include information about the chart type, title, axis ranges, and labels. If possible, describe statistics, extrema, outliers, correlations, point-wise comparisons, and trends for each plotted variable. If possible, briefly explain domain-specific insights, current events, and socio-political context that explain the data. Be concise and limit your response to {max_tokens} tokens.`

To incorporate heuristic alt text and markdown data tables, we include the following line between the example descriptions and instructions about description content: `You already know the following information about this figure and its data: '{generated heuristic alt text, possibly containing a table}'.`

By default, we also prompt models to describe `colors, sizes, textures`, but removed this clause for our evaluation to align with crowdsourced VisText captions.

4. N-gram similarity between generated and reference captions

In this section we measure BLEU, Rouge, and CHRF n-gram similarity between our generated and reference captions for VisText figures. Table 7 summarizes our results. We find that VL-T5 models typically have higher n-gram overlap with the human reference than GPT4-turbo, while our turbo + heuristic methods have higher Rouge-1 and Rouge-L recalls. This indicates turbo + heuristic descriptions contain overall more tokens from crowdsourced text compared to turbo and turbo + table methods, but do not emulate the style and formatting of the references as well as the finetuned VL-T5 models.

5. Similarity between shuffled images and descriptions

To measure whether similarity metrics from our evaluation are biased towards specific generation strategies, we compute each score using random pairs of alt text and references. For our comparisons to crowdsourced captions in §5.2, we randomly sample references for each MatplotAlt and VL-T5 description. For our measurement of similarity between alt texts and images using BLIP in §5.3, we select corresponding images randomly. A higher score in this setting indicates that the metric is biased towards text from that generation strategy regardless of whether its content is aligned to the figure.

We report all shuffled scores between VisText descriptions and

<table>
<tr><th>VisText Example Alt Text</th><th>Matplotlib Gallery Example Alt Text</th></tr>
<tr><td>
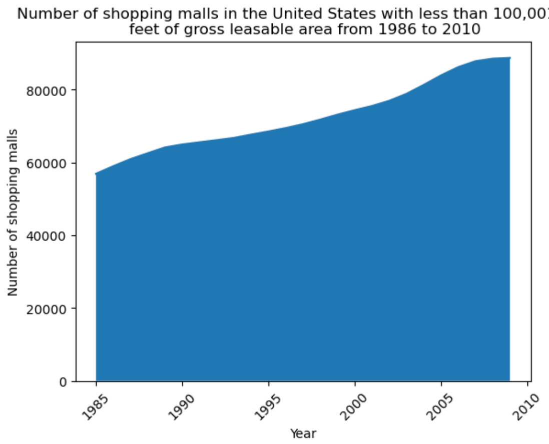

</td><td>
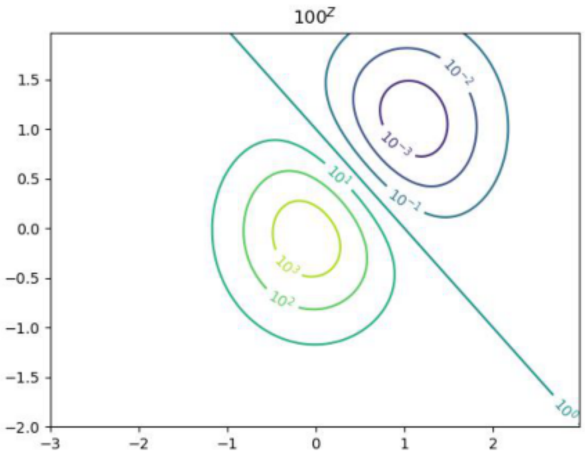

</td></tr>
<tr><td>

turbo description: This is an area chart titled "Malls in the United States with less than 100,001 sq. feet of gross leasable area." [*Label error*] It plots the number of shopping malls on the vertical y-axis, which ranges from 0 to 80,000 in increments of 20,000. The years are plotted on the horizontal x-axis from 1985 to around 2010. The chart shows a noticeable increase in the number of shopping malls over time, starting at about 20,000 malls in 1985 and rising to just under 80,000 by 2010 [*Value error*]. There's a steady growth up to around 1995, after which the rate of increase becomes more gradual, reaching a plateau in the mid 2000s before slightly decreasing towards 2010. [*Trend error*]

</td><td>

Heuristic description: A contour plot titled '100^z'. The x-axis ranges from -3 to 3 and the y-axis ranges from -2 to 2, both using linear scales. 7 contour lines are plotted with values 10^{-3}, 10^{-2}, 10^{-1}, 10^0, 10^1, 10^2, and 10^3. The max contour is centered around (-0.06283, -0.05828). [*Missing data context:* lacks description of contour shape including the diagonal line and minimum]

</td></tr>
<tr><td>

turbo + table description: This is an area chart showing the number of shopping malls in the United States with less than 100,001 square feet of gross leasable area over time. The horizontal x-axis ranges from the year 1985 to 2010, while the vertical y-axis represents the number of shopping malls from 0 to 80,000. The chart shows a substantial increase in the number of small malls from 1985, peaking between 2000 and 2005 [*Identity error*], and then a plateau or slight decrease towards 2010. There are no clear outliers, and the trend shows overall growth in mall numbers over time, with a stabilization or minor decline in the latter part of the series. [*Missing data context:* lacks chart values, e.g. number of malls in 1985]

</td><td>

turbo description: This is a contour plot with labels indicating different levels, likely representing a mathematical function's value over a two-dimensional space. The horizontal x-axis ranges from -3 to 3, while the vertical y-axis ranges from -2 to 2. Contour lines are presented with four distinct levels, indicated by 10^ 2, 10^ 1, 10^0, and 10^1 [*Axis error*: z axis contours extend from 10^ 3 to 10^3], each represented in a different color from yellow (innermost) to dark blue (outermost). A cyan diagonal line, annotated as 10^2, suggests an additional function or constraint cutting across the plot. The title is partially visible, reading only "100z." The plot shows a symmetrical pattern about the origin, with each contour line indicating a constant value. [*Deceptive error*: title is fully visible and pattern is symmetric around the diagonal line, not the origin]

</td></tr>
<tr><td>

turbo + heuristic description: This is an area chart titled 'Number of shopping malls in the United States with less than 100,001 sq. feet of gross leasable area from 1986 to 2010'. The year is plotted on the horizontal x-axis from 1980 to 2015, and the number of shopping malls is plotted on the vertical y-axis from 0 to 100,000, both using linear scales. The chart shows one variable plotted over time. The data exhibits a strict increasing trend from the year 1985, where the number of shopping malls is at its minimum of 56,910, peaking at its maximum in the year 2009 with 88,760 shopping malls. There are no apparent outliers, and the strict increase indicates a consistent growth in the number of shopping malls with fewer than 100,001 sq. feet of leasable area over the given time period. After reaching the peak in 2009, there seems to be a slight plateau or very gradual decline towards 2010, though the data does not extend beyond that year to visualize any further trends.
[We consider this alt text correct]

</td><td>

turbo + heuristic description: This contour plot is titled '100^z' and visually represents three-dimensional data on a two-dimensional plane. The x-axis is labeled and ranges from -3 to 3, while the y-axis ranges from -2 to 2. There are seven contour lines corresponding to values 10^{-3}, 10^{-2}, 10^{-1}, 10^0, 10^1, 10^2, and 10^3. These lines indicate the points on the plane where the function 100^z has the same value. The contour values are increasing as they move towards the center, with the max contour centered approximately at (-0.06283, -0.05828). The colors transition from green (lower values) to yellow (higher values) and then to blue for the highest values towards the center of the plot. [*Deceptive error:* Blue/purple colors represent lowest values] The plot lacks a legend, but the contour values are labeled directly on the lines. The overall trend shows that [*Cutoff*]

</td></tr>
</table>

Table 6: *Example descriptions containing errors for one VisText figure, and one Matplotlib gallery figure. Errors are colored and tagged by type.*

VisText Alt Text Type	BLEU	Rouge-1 F1	Rouge-1 recall	Rouge-2 F1	Rouge-2 recall	Rouge-L F1	Rouge-L recall	CHRF
VL-T5 - image only	5.504	33.28	30.91	13.01	12.14	24.09	22.43	26.36
VL-T5 - image + scene graph	**29.47**	**59.83**	52.03	**43.55**	**37.88**	**49.29**	42.90	**50.59**
VL-T5 - image + table	**29.45**	**60.30**	51.90	**43.93**	**37.83**	**49.75**	42.86	**50.89**
Heuristic	15.56	54.60	58.37	32.51	35.04	39.56	42.50	45.89
turbo	14.14	46.37	63.77	22.61	31.47	32.32	44.77	46.45
turbo + table	13.46	46.06	66.00	22.35	32.47	31.97	46.23	46.62
turbo + heuristic	15.66	48.37	67.11	25.97	36.52	35.00	**48.96**	49.22
turbo + table + heuristic	15.02	47.55	**67.68**	25.50	36.84	34.32	**49.26**	48.94

Table 7: *N-gram similarity scores between crowdsourced and generated descriptions for each method. Higher is better for each metric. VisText VL-T5 models typically have higher n-gram overlap with the human references, while turbo + heuristic generations have higher Rouge-2 and Rouge-L recalls.*

crowdsourced references in Table 8. All token n-gram overlap F1 scores decreased substantially in this setting except for VL-T5 - imageonly, suggesting that these similarity metrics are not biased towards any one generation method. However, CHRF, Rouge-1 recall, BertScore recall, and BLEURT are all consistently higher for MatplotAlt methods, possibly due to their longer generations and the higher fluency of GPT4 generations.

Our shuffled BLIP results are in Table 9. Matching figures and descriptions were overwhelmingly scored higher than the random pairs, with an average difference of 0.60 in probability and 0.12 in cosine similarity. On the gallery dataset, we find that the shuffled turbo captions scored the worst, indicating that its high score on the matching pairs is not due to a preference for GPT4-generated texts.

6. Correlation Between Evaluation Metrics and Errors

We measure correlations between each observed error type and evaluation metrics, including description length, n-gram and BERT-based similarity to human references, and BLIP scores between images and captions. Table 10 summarizes our results averaged over both VisText and Matplotlib Gallery datasets and each of our Heuristic and turbo methods.

On one hand, we find that several of the error types have no significant ($p > 0.005$) correlations with our other evaluation metrics including axis, value, trend, number name, and nonsense. This suggests that even captions with high similarity to a human-written reference or image embedding may contain these errors. On the other, several error types are correlated to many of the other metrics, including label, cutoff, and deceptive. Description length is also correlated to several of the caption similarity metrics, particularly their precision components. This is not surprising as longer descriptions likely contain words and meanings not present in the shorter human references, and confirms that the n-gram matching metrics are likely not as relevant due to this discrepancy in lengths. At the same time, Rouge2 F1 has the highest correlation to captions being labeled as correct or value-correct, suggesting that length and word overlap still play a significant role in our manual evaluation of correctness.

VisText Alt Text Type	BLEU	Rouge-1 F1	Rouge-1 recall	Rouge-2 F1	Rouge-2 recall	Rouge-L F1	Rouge-L recall	CHRF	BertScore F1	BertScore recall	BLEURT
VL-T5 - image only	**4.372**	31.29	29.23	11.33	**10.64**	**22.90**	21.45	25.09	**0.8565**	0.8499	-1.084
VL-T5 - image + scene graph	4.059	29.90	26.08	**11.57**	10.12	21.54	18.83	23.55	**0.8559**	0.8458	-1.009
VL-T5 - image + table	4.063	30.05	25.89	**11.88**	10.26	21.75	18.76	23.76	**0.8564**	0.8458	-1.007
Heuristic	3.109	**34.51**	36.77	10.35	**11.05**	21.47	22.99	27.86	0.8458	**0.8520**	-0.8991
turbo	2.170	28.48	39.18	5.909	8.227	17.78	24.62	28.86	0.8440	**0.8522**	**-0.7965**
turbo + table	2.116	28.13	40.24	5.868	8.504	17.53	25.27	28.80	0.8424	**0.8522**	**-0.7944**
turbo + heuristic	2.621	29.64	40.89	7.541	10.53	18.59	**25.85**	**29.88**	0.8455	**0.8551**	-0.8234
turbo + table + heuristic	2.510	29.23	**41.46**	7.419	**10.63**	18.29	**26.13**	**29.79**	0.8443	**0.8549**	-0.8291

Table 8: *Similarity scores for non-matching shuffled pairs of VisText crowdsourced captions and MatplotAlt-generated descriptions.*

Dataset	Alt Text Type	Matching Prob.	Cosine Sim.
VisText	Human	0.9999 / 0.3166	0.4992 / 0.3570
VisText	Heuristic	0.9999 / 0.3127	0.5037 / 0.3576
VisText	turbo	0.9985 / 0.2653	0.5039 / 0.3675
VisText	turbo + heuristic	0.9999 / 0.3282	0.5079 / 0.3677
VisText	turbo + table	0.9977 / 0.2726	0.5050 / 0.3697
VisText	turbo + heuristic + table	0.9999 / 0.3170	0.5081 / 0.3678
Gallery	Heuristic	0.9270 / 0.5925	0.4263 / 0.3652
Gallery	turbo	**0.9766** / 0.4897	**0.4879** / 0.3883
Gallery	turbo + heuristic	0.9629 / 0.5241	0.4717 / 0.3816

Table 9: *BLIP scores for matching / shuffled pairs of MatplotAlt descriptions and images from each dataset.*

Len. & error type → Correlated metric ↓	Description length	Correct	Value-correct	CTE	AE	VE	IE	TE	LE	MC	UC	R	C	NS	DE	NE
Description len.	N/A	−0.131	−0.110	-	-	-	-	-	0.150	−0.161	-	**0.193**	**0.277**	-	0.201	-
BLEU	−0.233	0.191	0.162	**−0.206**	-	-	-	-	-	-	-	-	-	-	-	-
Rouge1 (f1)	−0.523	0.165	-	-	-	-	-	-	−0.174	-	-	-	−0.148	-	−0.128	-
Rouge1 (p)	−0.660	0.133	-	-	-	-	-	-	−0.191	-	-	-	−0.175	-	−0.151	-
Rouge1 (r)	0.397	-	-	-	-	-	-	-	-	-	-	-	0.138	-	-	-
Rouge2 (f1)	−0.534	**0.242**	**0.218**	-	-	-	-	-	−0.216	-	-	-	-	-	**−0.220**	-
Rouge2 (p)	−0.688	0.211	0.188	-	-	-	-	-	−0.233	-	-	-	−0.132	-	**−0.221**	-
Rouge2 (r)	-	0.167	0.164	-	-	-	-	-	-	−0.131	-	-	-	-	-	-
RougeL (f1)	−0.535	0.200	0.178	-	-	-	-	-	**−0.269**	-	-	-	−0.144	-	**−0.219**	-
RougeL (p)	**−0.713**	0.166	0.142	-	-	-	-	-	−0.257	-	-	-	−0.182	-	−0.211	-
RougeL (r)	0.241	-	-	-	-	-	-	-	-	-	**0.144**	-	-	-	-	-
CHRF	0.052	0.193	0.136	−0.155	-	-	-	-	-	−0.207	-	-	-	-	-	-
BertScore (f1)	−0.298	0.144	-	-	-	-	-	-	−0.207	-	-	-	−0.192	-	−0.158	-
BertScore (p)	−0.428	-	-	-	-	-	-	-	−0.188	-	-	-	−0.262	-	−0.138	-
BertScore (r)	-	0.127	-	-	-	-	-	-	−0.129	-	-	-	-	-	-	-
BLEURT	−0.043	-	-	-	-	-	-	-	−0.142	-	-	-	-	-	-	-
BLIP matching prob.	-	0.126	-	-	-	-	-	-	-	−0.140	-	-	−0.117	-	-	-
BLIP cosine sim.	-	**0.237**	-	0.110	-	-	**−0.166**	-	-	**−0.239**	-	−0.147	−0.126	-	-	-

Table 10: *Correlations between description length, similarity metrics, and each error type in annotated alt texts. Dashes indicate insignifigant correlations with p-values greater than 0.005.*
